# Cell signaling and transcription factor genes expressed during whole body regeneration in a colonial chordate

**DOI:** 10.1186/1471-213X-8-100

**Published:** 2008-10-12

**Authors:** Yuval Rinkevich, Baruch Rinkevich, Ram Reshef

**Affiliations:** 1 Faculty of Biology, Technion – Israel Institute of Technology, Haifa, Israel; 2 Israel Oceanographic and Limnological Research, National Institute of Oceanography, Tel Shikmona, Haifa 31080, Israel

## Abstract

**Background:**

The restoration of adults from fragments of blood vessels in botryllid ascidians (termed whole body regeneration [WBR]) represents an inimitable event in the chordates, which is poorly understood on the mechanistic level.

**Results:**

To elucidate mechanisms underlying this phenomenon, a subtracted EST library for early WBR stages was previously assembled, revealing 76 putative genes belonging to major signaling pathways, including *Notch/Delta*, *JAK/STAT*, protein kinases, nuclear receptors, Ras oncogene family members, G-Protein coupled receptor (GPCR) and transforming growth factor beta (TGF-β) signaling. RT-PCR on selected transcripts documented specific up-regulation in only regenerating fragments, pointing to a broad activation of these signaling pathways at onset of WBR. The followed-up expression pattern of seven representative transcripts from *JAK/STAT *signaling (*Bl-STAT*), the Ras oncogene family (*Bl-Rap1A, Bl-Rab-33*), the protein kinase family (*Bl-Mnk*), *Bl-Cnot*, *Bl-Slit *and *Bl-Bax inhibitor*, revealed systemic and site specific activations during WBR in a sub-population of circulatory cells.

**Conclusion:**

WBR in the non-vertebrate chordate *Botrylloides leachi *is a multifaceted phenomenon, presided by a complex array of cell signaling and transcription factors. Above results, provide a first insight into the whole genome molecular machinery of this unique regeneration process, and reveal the broad participation of cell signaling and transcription factors in the process. While regeneration involves the participation of specific cell populations, WBR signals are systemically expressed at the organism level.

## Background

Whereas most animal phyla share the capability to repair damaged tissues and some organs, whole body reconstitution from small fragments is a rare event in deuterostomes and is mostly confined to protostomes like sponges [1], cnidarians [2] and flatworms [3]. An exception is the dramatic botryllid ascidians phenomenon of whole-body regeneration (WBR) from minute vasculature fragments [4-8], best studied in *B. leachi*. As the capacity for wide-range regeneration is limited in chordates [9], WBR may be used as a model system for studying the restricted regeneration ability and the basic mechanisms underlying vertebrate regeneration.

**Figure 1:**
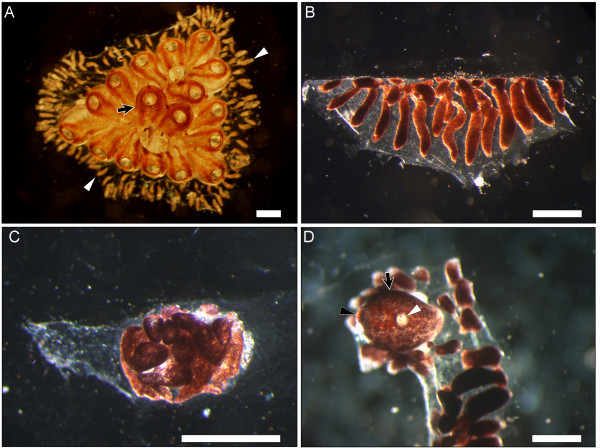
**Whole body regeneration (WBR) in the colonial chordate *Botrylloides leachi***. **A**: A *B. leachi *colony with numerous clonal modules; zooids (arrow). Zooids are embedded in a gelatinous sheet of tunic and are interconnected by a network of blood vessels, which terminates in hundreds of blind ampullar termini (arrowheads). **B**: An experimental fragment of marginal blood vessels immediately after separation from the colony. **C**: A vessel mass created after seven days, from the aggregation and migration of blood vessels within the tunic embedment. **D**: A single adult zooid (black arrow) regenerates from the marginal blood vessels after 10–14 days post separation from *B. leachi *colonies. Regenerated zooids are reproductive and are fully functional with incurrent (black arrowhead) and outcurrent (white arrowhead) siphons. Scale bar = 500 μm.

The Mediterranean urochordate *B. leachi *(Fig. 1a) is a common shallow water encrusting colonial sea squirt [10]. Each colony is composed of few to thousands of genetically identical modules (zooids; Fig. 1a, arrow), each 2–3 mm long, embedded within a semi-translucent gelatinous matrix, the tunic. Zooids are arranged in systems of two parallel long, often serpentine, rows connected to each other through a delicate, one cell thick blood vessel network [11]. Numerous pear-shaped extensions (ampullae) stretch from the ramifying vasculature toward the colony margins (Fig. 1a, arrowheads).

WBR in *B. leachi *develops in areas deprived of zooids such as marginal ampullae (Fig. 1b). In fact, even a single ampulla, containing approximately 100–200 blood cells, is capable of regenerating a whole functional organism within 10–14 days from isolation [6-8]. Through highly coordinated processes of vessels' movement, anastomoses and coalescence within the tunic matrix, an opaque mass of cells is formed at 'selected' tunic spots (Fig. 1c), along with a new circulatory system that is gradually being established. Three distinctive morphological phases for WBR were identified on the cellular level [8]. Phase I (days 1–3) entails the formation of a new microenvironment through restructuring of the vessel architecture. Morphologically, this phase involves the detachment of vascular epithelium from the tunic embedment, followed by cellular divisions in the vascular epithelium, which creates dozens of small compartments ('regeneration niches') within vasculature lumens. Therefore, while in other epimorphic regeneration systems restoration starts with the formation of a transient structure termed blastema, the *B. leachi *WBR is noted by *de-novo *structuring of regeneration niches. Phase II (days 3–5) is characterized by homing of lymphocyte-like cells, their aggregation within regeneration niches and their subsequent proliferation. Yet, nothing is known regarding the cell origin and the homing process of these cells. Morphologically, multiple foci of regeneration are initiated simultaneously. This distinguishes the *B. leachi *WBR from most other regeneration model systems studied, where normally only a single restoration center is formed. During phase III (days 5 and thereafter), the race for predominance between collectives of foci (in a single experimental fragment) culminates in the maturation of only a single bud to a functional filter-feeding adult ascidian-zooid equipped with an incurrent (Fig. 1d, black arrow) and outcurrent siphon (Fig 1d, white arrowhead). This system is therefore the first documented case of WBR reveals the capability of restoring not only the soma, but also the germ line [6].

The exact cellular and molecular mechanisms of *B. leachi *WBR in which the entire urochordate body plan is formed from a few somatic cells have yet to be well understood. Elucidating the molecular schemes could revolutionize our understanding concerning the nature of regeneration and functions of pluripotent adult stem cells (lymphocyte-like cells) in WBR and other regeneration processes. In *B. leachi*, as in many other marine organisms with yet poorly established genetic backgrounds, the ease and reproducible methodology of expressed sequence tags (ESTs) can be employed for generating reliable molecular profiles of multi-gene expressions underlying the complex WBR process [12]. By focusing on a library of ESTs originated from early stages of *B. leachi *WBR (2–4 days following isolation of peripheral ampullae; phases I and II [8]), we wish to address one facet of the molecular machinery governing WBR, the cell signaling and transcription factor profiles. This stems from previous observations [8,14], which imply that signaling pathways could be common themes in initiating epimorphic regeneration events in multicellular organisms.

## Results

### EST library of whole body regeneration reveals multiple putative signaling pathway components

We previously assembled a normalized and subtracted EST library, enriched with genes expressed in early stages of *B. leachi *whole body regeneration (WBR) (up to 4 days following ampullae isolation; [13]). Because of previous findings [8,14], of particular relevance to us are 16.7% of the transcripts (n = 76; see Additional file 1) which are significantly similar to known genes involved in cell communication and signaling. The relatively high profile level of putative genes functioning in cell signaling and transcription points to genome-wide involvement of signaling processes at onset of *B. leachi *WBR.

Of the 76 unique putative cell-communication and signaling molecules (see Additional file 1), 9 transcripts have multiple representations (contigs) and 67 are singlets, all potentially protein-coding transcripts. This suggests that the putatively rich WBR transcriptome has not been yet thoroughly sampled and that high copy transcripts were not manifested, implying that normalization was highly effective. Only matches with less than a 2.0e-5 chance occurrence probability were classified as significant. In general, the identified transcripts revealed a broad range of functional categories with alleged roles in diverse biological processes including cell signaling, chemotaxis and cell determination (see Additional file 1). These 76 transcripts represent putative components of major signaling pathways, including nuclear receptors (see Additional file 1, seq. 24, 33, 76), protein kinase (seq. 8, 20, 26, 54), Notch/Delta (seq. 2, 75), JAK/STAT (seq. 39), G-Protein coupled receptors (GPCR, seq. 3, 19, 29, 40) transforming growth factor beta (TGF-β, seq 13) and others, illustrating a complex profile of signaling systems activated during early phases of *B. leachi *WBR.

Domain search that was carried out on all sequences revealed the broad presence of conserved characteristic domains (data not shown) sustaining the predicted function of the proteins in signaling responses. For example, domain search of *Notch *transcripts revealed ankyrin repeats (E-value 7e-23), mediating protein-protein interactions, a NOD (NOTCH protein domain; E-value 1e-10), NODP domains present in many NOTCH proteins from multiple species, and an EGF-like domain (E-value 9.90E-07), all present in a large number of membrane-bound and extracellular proteins (mostly animals). Domain search on STAT protein identified STAT binding domain, the characteristic DNA binding domain of STAT proteins. Domain search on protein kinase family members identified a catalytic domain (STKc domain, E-value 1e-73) belonging to serine or threonine-specific kinase family members featuring catalytic and activation loops, an ATP binding pocket, a substrate binding pocket, and a CNH Domain (E-value 9e-23) found in NIK1-like kinases.

### Temporal expression of signaling genes during WBR

**Figure 2:**
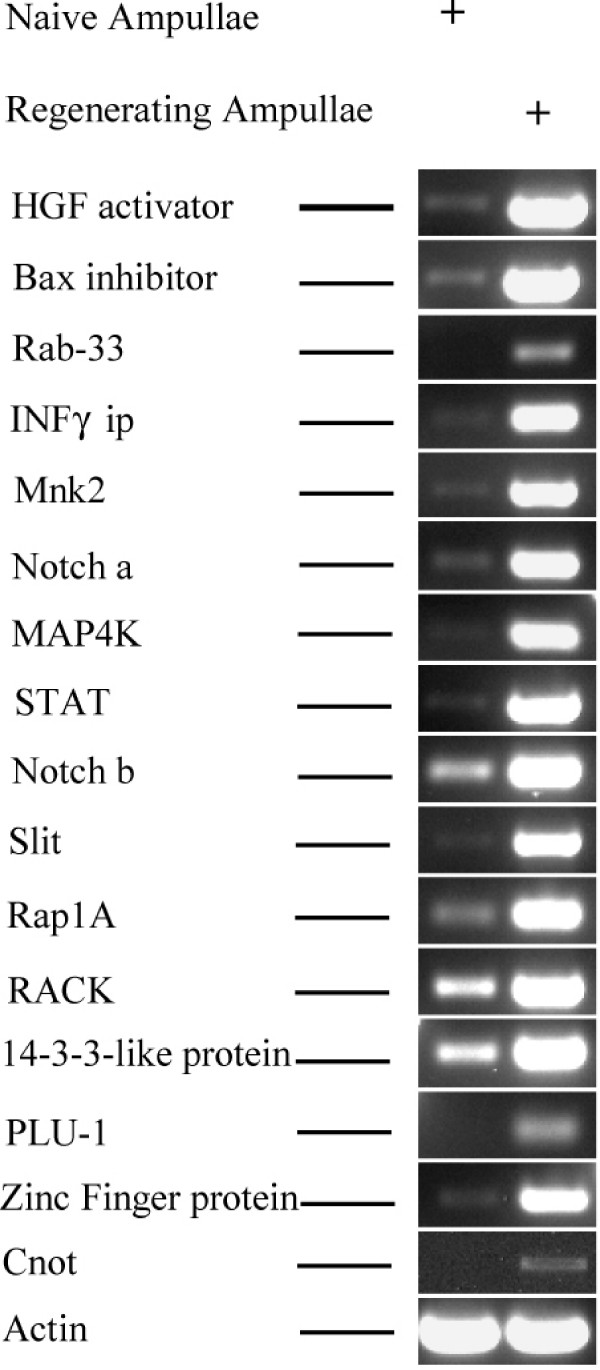
**Temporal expression of representative signaling transcripts during WBR**. Specific primers (Table 1) were used to amplify cell communication and signaling-related transcripts during WBR. Representative transcripts include *hepatocyte growth factor *(*HGF*) *activator*, *Bax inhibitor*, *Rab-33*, *interferon gamma *(*INFγ*) *inducible protein*, *MAP kinase signal-integrating kinase 2 *(*Mnk2*), two different receptor protein *Notch *transcripts (*Notch a, Notch b*), *Slit*, *Rap1A*, receptor for activated PKC (*RACK*), PKC inhibitor protein eta (*14-3-3-like protein*), transcription factors *PLU-1*, Zinc Finger domain protein and *Cnot*. Temporal expressions include naïve ampullae and early stages of regeneration (2–4 days) presumably trapped in our EST library. The cytoplasmic actin bands at the bottom serve as a positive control.

In order to confirm the specificity of the EST library to WBR and determine the exclusive up-regulation of transcripts during *B. leachi *WBR, series of RT-PCR reactions were performed on mRNAs from intact ampullae and incised ampullae at onset of WBR (Fig. 2), using sequence specific primers (Table 1). Sixteen transcripts encoding for putative proteins with diverse functions were chosen from the list, based on their roles as key signaling pathway components (see discussion). The list of chosen transcripts included genes repeatedly present (as contigs) in the EST library, such as *Bax inhibitor *(3 contigs) and Transmembrane receptor *Notch *(8 contigs), as well as singlet category genes including *HGF activator*, *Rab-33 *oncoprotein, *Interferon gamma inducible protein*, *Mnk *and *MAP4K*, and others. As expected, the RT PCR analysis (Fig. 2) revealed specific transcript up-regulation during *B. leachi *WBR; low or no expression in intact ampullae compared to elevated expressions in regenerating ampullae at early WBR stages (2–4 days post isolation), suggesting the involvement of the signaling transcripts in the restoration of zooids in *B. leachi*.

**Table 1:** Primers used for RT-PCR amplification of putative cell signaling transcripts during *B. leachi *WBR.

Genebank Accession No	Homologue name	5' primer	3' primer	Seq. length
EW713308	STAT	5' AGACTCAGCTCCGCGTTTG 3'	5' AATCATTTGGCAGAACGGAC 3'	454 bp
EW713339	Slit	5' GGTTAATCGGCAGTCGCAAAG 3'	5' GGCCTCAATAAATTACGTGTGC 3'	453 bp
EW713329	Rap1A	5' CTGTTAGGGCTGAGCATCATAG 3'	5' TGGCAAGACAATGGAATAACTG 3'	174 bp
EW713278	14-3-3	5' ACGTTGCGGAAGTGATAGACAG 3'	5' ATGGCATCATCGAAAGCTTC 3'	217 bp
EW713279	Zinc finger	5' AGGCCTTCCAATCCATCC 3'	5' GGTGTTCCGCTTCCGTTTG 3'	266 bp
EW713331	Plu-1	5' TCGTATTCGAACTGGCAATTTC 3'	5' CTACAGGGAAGAGCAGGTTGC 3'	137 bp
EW713289	Mnk2	5' ATGTGGTGAGGATTGTGGATG 3'	5' AATCGTGAATGTGGCTGAAGAC 3'	475 bp
EW713295	MAPKKKK	5' AGTCGTTGTGCTCTCTTGTGC 3'	5' CCCAAACACGGAAGGAATG 3'	234 bp
EW713334	Rab-33	5' CGCAATATTAGTGTCCACATGG 3'	5' TCCTACTCGAACTGAAGCCAC 3'	316 bp
EW713275	Bax inhibitor 1	5' AAATGAAATCTATGGCACAGCG 3'	5' TGACTGCCTTTATGACAACCTC 3'	578 bp
EW713320	HGF activator	5' CCCACGTTATGAGCGCTTCTTC 3'	5' ACCATTCCCTCTCCCGTCAAC 3'	509 bp
EW713287	Cnot	5' TTCTCCGTAACGATTCAACTTG 3'	5' GGGTATTTGTCATGTTTCTTGC 3'	469 bp
EW713277	Notch a	5' CATGAACCTAGTGTCGCACTCG 3'	5' GTGTGGATCACGTGTTGGG 3'	260 bp
EW713344	Notch b	5' TGTGAGTCCCAAGTCGTGAATC 3'	5' AAAGCTCGCGCTGCAACTAC 3'	225 bp
EF125177	Cytoplasmic actin	5' GAAATCGTGCCGTGACATCAAAG 3'	5' GCGGTGATTCCCTTCTGCATAC 3'	338 bp

### Expression-patterns of representative cell signaling genes and transcription factors during WBR

To further elucidate the participation of EST library expressed genes in WBR, we analyzed the spatio-temporal expression patterns of seven representative signaling transcripts from the list (see Additional file 1). This was done by conducting *in situ *hybridizations on paraffin embedded tissue sections of: *Bl-Rap1A*, *Bl-Cnot*, *Bl-Slit*, *Bl-STAT *(signal transducer and activator of transcription), *Bl-Rab-33*, *Bl-Bax inhibitor *and *Bl-Mnk *(MAP kinase integrating kinase) overall representing the JAK/STAT signaling pathway, the Ras oncogene family and the protein kinase family. Since we do not have a full open reading frame for these seven genes, their gene ascription remains putative.

**Figure 3:**
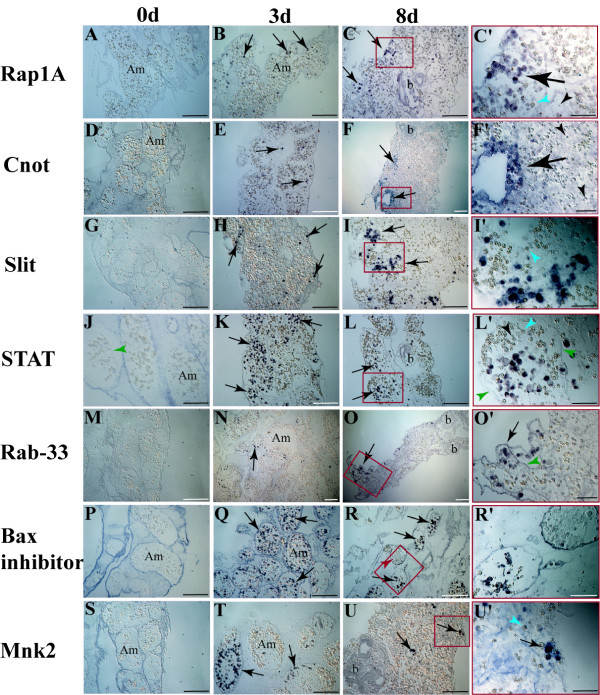
**Expression-patterns of representative signaling transcripts during WBR**. Whole-mount RNA *in situ *hybridization of *Bl-Rap1A *(A-C'), *Bl-Cnot *(D-F'), *Bl-Slit *(G-I'), *Bl-STAT *(J-L'), *Bl-Rab-33 *(M-O'), *Bl-Bax inhibitor *(P-R') and *Bl-Mnk *(S-U') on colonial, naïve and regenerating blood vessels at three and eight days post separation. Naïve ampullae of *B. leachi *colonies show no detectable staining of transcripts within blood cells (A, D, G, J, M, P, S). During WBR, specific staining of the transcripts is present within regenerating fragments in a population of large (12–15 μm) round cells, and is absent from morula cells (I', L', U' blue arrowheads) and lymphocyte-like cells (C', F', L', black arrowhead). *Bl-Rap1A, Bl-Cnot *and *Bl-Slit *at three days of regeneration, stained circulatory cells (B, E, H respectively, arrows) juxtaposed to the vessel epithelium (can be observed in J, L', O', green arrowheads). At eight days of regeneration, staining was present in cell conglomerates within peripheral sites (C, F, I arrows) and absent from regenerating buds (C, F). *Bl-STAT *and *Bl-Rab-33 *at three days of regeneration, stained cells within multiple niches (K, N respectively, arrows). At eight days of regeneration, staining is still absent from developing buds and their niches (L, O) but present within niches in proximity to vessel epithelium (L', O' green arrowheads). *Bl-Bax inhibitor *and *Bl-Mnk *are expressed differentially within niches. At three days of regeneration, *Bl-Bax inhibitor *is systemically expressed within most niches (Q, arrows), but at eight days of regeneration, is completely absent from degenerating niches (R, red arrowhead). At three days, *Bl-Mnk *displays a comprehensive expression within selected niches (T, arrows), while absent from neighboring sites. Later, *Bl-Mnk *expression is confined to small cell aggregates within distant niches (U, arrows) and absent from developing buds (U). Red rectangles in 8 d column correspond to the respective enlargements. Am, ampullae; b, bud. Scale bar represents 100 μm.

An overview of the expression patterns of these seven transcripts is depicted in Fig. 3. The *in situ *hybridization analyses examined three stages of regeneration: day 0 (controls), we used intact ampullae attached to *B. leachi *colonies, in which expressions were assessed along blastogenic cycles A-C (Fig. 1A), as well as marginal ampullae immediately after separation from *B. leachi *colonies (Fig. 1B). Day three represented phase I of WBR in which lacunae of blood vessels are formed within the tunic embedment (Fig. 1C, sensu 8) and day eight represented phase III of WBR in which bud development and organogenesis is taking place (sensu 8).

Intact peripheral ampullae and centrally located blood vessels within *B. leachi *colonies (0 d) were not stained with any of the probes (Fig. 3A,D,G,J,M,P,S). In contrast, expressions of all seven transcripts were clearly documented in ampullae at day three and day eight of WBR, concurrent with bud development. In these preparations, we noticed a strong expression restricted to a morphologically distinct sub-population of large, round circulating cells (12–15 μm diameter). This sub-population of blood cells is uncommon in the colonial vasculature during regular blastogenic cycles A-C, but appears widespread throughout regenerating ampullae as from early stages of WBR (unpublished observations). The most frequently circulating cell types, morula cells (Fig. 3, blue arrowheads) and lymphocyte-like cells were not stained in the *in situ *hybridization sections.

At day three, *Bl-Rap1A*, *Bl-Cnot *and *Bl-Slit *displayed a similar expression pattern, found within conglomerates of circulatory cells within niches located at the periphery of the regenerating fragment (sensu 8), in juxtaposition to the blood vessel's epithelium (Fig. 3B,E,H, arrows, respectively). At regeneration day eight staining was not present in regenerating buds (b, for *Bl-Rap1A*, Fig. 3C; for *Bl-Cnot*, Fig. 3F, for *Bl-Slit*, data not shown), but appeared within distant sites in which no regeneration was observed (Fig. 3C,C',F,F',I,I', arrows, respectively). *Bl-STAT *and *Bl-Rab-33 *revealed a similar distribution pattern. At day three of WBR, preceding the formation of regenerating buds, staining was distributed systemically within most regeneration niches, in a subpopulation of large blood cells with both central and peripheral fragment locations (Fig. 3K,N, arrows, respectively). At day eight, *Bl-STAT *transcripts were generally located in cells inhabiting marginal niches (Fig. 3L,L', arrows) although few of them were documented in niches occupied by developing buds (Fig. 3L, arrowhead). At day eight, *Bl-Rab-33 *transcripts were restricted to distant niches (Fig. 3O,O', arrows) and were not documented in niches occupied by developing buds (Fig. 3O, arrowheads). *Bl-Bax inhibitor *and *Bl-Mnk *were expressed differentially within regenerating niches. *Bl-Bax *inhibitor showed at day three a ubiquitous expression within regenerating niches (Fig. 3Q, arrows), but at day eight was completely absent from niches undergoing degeneration (Fig. 3R, red arrowhead). *Bl-Mnk *displayed at day three a ubiquitous expression within only few niches (Fig. 3T, arrows). However, at day eight of regeneration, *Bl-Mnk *was confined to small cell aggregates within distant niches (Fig. 3U, arrows) and was absent from developing buds (Fig. 3U, arrowhead) similarly to the aforementioned transcripts. Specific sense probes for all seven studied transcripts were used as controls, revealing no detectable staining pattern (data not shown).

### Expression-patterns of representative cell signaling genes and transcription factors during stage D of blastogenesis

**Figure 4:**
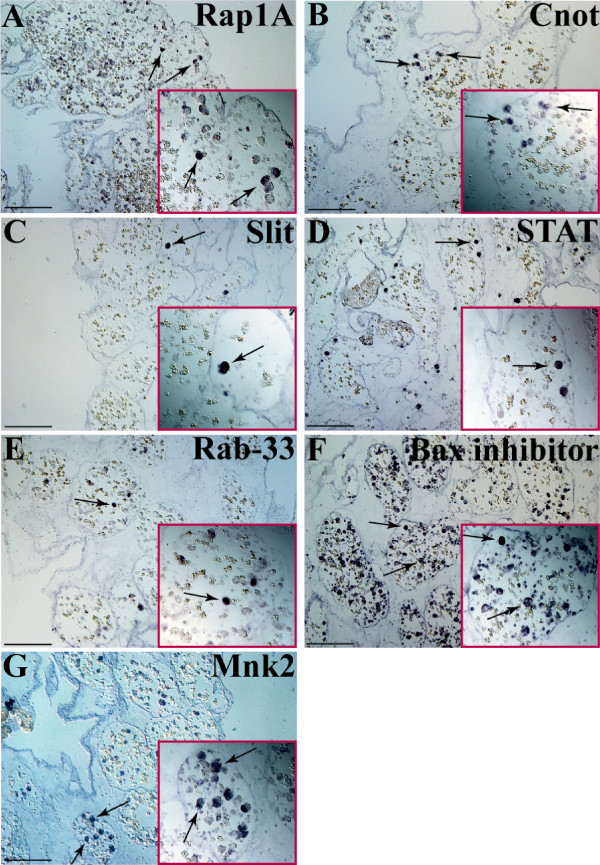
**Expression-patterns of representative transcripts in *B. leachi *during stage D of blastogenesis**. Whole-mount RNA *in situ *hybridization of *Bl-Rap1A *(A), *Bl-Cnot *(B), *Bl-Slit *(C), *Bl-STAT *(D), *Bl-Rab-33 *(E), *Bl-Bax inhibitor *(F) and *Bl-Mnk *(G) on blastogenic stage D (takeover) colonies. During this 24–36 h period, signaling transcripts are expressed within marginal ampullae in single cells (A-G arrows). Red rectangles represent enlargements of the arrows region. Scale bar represents 100 μm.

Blastogenesis involves a weekly and cyclical process (stage A-D) in which new zooids are developed from the body wall of parental zooids. This process culminates in a transient 24–36 h period (stage D) termed 'take-over' [15], in which parental zooid's tissues go through synchronized apoptosis and are gradually resorbed by circulating phagocytic cells concurrent with their replacement by a new generation of buds. Both humeral and cellular components of the vasculature network play an active role in coordinating and executing the 'take-over' process [16,17]. In contrast to blastogenic stages A-C in which no expression was observed in either transcripts (data not shown), in blastogenic stage D we observed many large round cells that were positively stained for the seven transcripts (Fig. 4A–G, arrows and enlargements), resembling that of early stages of WBR. Stained cells are preferentially localized to peripheral sites and are observed to adhere to epithelial walls of blood vessels.

## Discussion

The phenomenon of whole body regeneration (WBR) in botryllid ascidians is a poorly understood, rare phenomenon in chordates. This remarkable intricate developmental system sets the botryllid WBR assay [6-8], as one of the most appropriate model systems for studying evolutionary issues related to regeneration. In this paper, we studied further the molecular machinery governing WBR by focusing on cell signaling and transcription factors expressed at the onset of *B. leachi *WBR (3–8 days). We characterized 76 genes, representing signaling pathways such as TGF-β, receptor tyrosine kinase (RTK), Notch/Delta, JAK/STAT, nuclear receptors, G-protein coupled receptors (GPCR) including Ras oncogene and zinc-finger family members and revealed the expression patterns of seven representative transcripts during regeneration and colony astogeny. These transcripts include members of JAK/STAT signaling (*Bl-STAT*), the Ras oncogene family (*Bl-Rap1A, Bl-Rab-33*) and the protein kinase family (*Bl-Mnk*), as well as *Bl-Cnot*, *Bl-Slit *and *Bl-Bax inhibitor*. Expressions of the transcripts were restricted during regeneration to a sub-population of large circulating blood cells, which also appeared, in smaller numbers, at blastogenic stage D. The exact nature and lineage of this cell population awaits further investigation as they appear as a central cellular component to *B. leachi *WBR. However, the systemic expression of these transcripts throughout regenerating fragments at early stages (Fig. 3, day 3), illustrates at the molecular level the systemic induction profile proposed earlier [8], following the documentation of multiple buds regenerating within a single isolated fragment. We also noticed that stage D inaugurates the idiotypic molecular signature of early WBR process (Fig. 4A–G), implying that the molecular mechanisms underlying early stages of WBR are analogously activated during colony astogeny, at the takeover (blastogenic stage D) phase.

During early stages of WBR, circulating cells are recruited to specific sites where regeneration niches are formed. These totipotent cells aggregate and subsequently proliferate in these developmental sites [8]. *Rap1A*, a member of the Ras oncogene family, is a positive regulator of T cell behavior through its roles in directing integrin activation and augmenting lymphocyte responses [18]. *Rap1A *suppression inhibits cell adhesion [19-21], while it's constitutively active form is sufficient to induce integrin mediated cell adhesion or cadherin-mediated adhesion [22]. Following the roles played by *Rap1A*, we suggest that the putative *Bl-Rap1A *may function directly in selectively recruiting specific circulating cell types to active areas of regeneration through an adherence-based mechanism, thereby facilitating their conglomerate and subsequent activation. Following the same line, *Slit *has an evolutionarily conserved role as a guidance molecule in directing the migration of diverse cell types during embryonic development [23,24] cancer [25] and immunity [26]. The idiotypic expression patterns of *Bl-Rap1A *and *Bl-Slit *(Fig. 3C,I) suggest a similar role in the recruitment of particular cells during WBR to regenerating sites (at early stages) and degenerating sites (at later stages, day 8 in this work, and at blastogenic stage D).

*Bax inhibitor *is an intracellular multi-membrane-spanning protein, originally identified for its evolutionarily conserved function in suppression of programmed cell death by hindering *BAX*'s pro-apoptotic functions [27]. During WBR, *Bl-Bax inhibitor *is expressed systemically at very early stages (Fig. 3Q) and at day eight its expression is confined to several regeneration niches (Fig. 3R, arrows), while absent from others (Fig 3R, arrowhead). WBR involves a unique strategy in which the development of numerous regenerative sites is followed by only a single mature bud [8]. *Bl-Bax inhibitor *may thus play a protective role within regenerating niches or be a part of the mechanism underlying the selection between regenerating and degenerating niches. During stage D of blastogenesis, massive apoptosis occurs in the colony, within parental zooid's tissues and within 20%–30% of circulating haemocytes [28,29] during which the proapoptotic *Bax *protein is also documented [30]. Our finding of the expression of *Bl-Bax inhibitor *within circulatory cells (Fig. 4F) during this stage is an important and intriguing observation, as it is commonly believed [16,17] that blastogenic stage D represents a 'destruction' cell death phase, not a rejuvenilization process.

Regeneration of organs and tissues is a widespread phenomena in the animal kingdom, revealing diverse strategies and varying roles of different cell types [14]. Within this array of regeneration phenomena, the *B. leachi *WBR system is fundamentally different from other model systems for regeneration by key properties at the morphological, cellular and mechanistic levels [8]. On the other hand, the outcomes of this and earlier study [13] point toward widespread and shared molecular pathways between *B. leachi *WBR and other regenerating model systems [14,31-33]. In this regard, it appears that only a few molecular cascades partake in most of the developmental pathways in the animal kingdom [34-36]. Cell signaling pathways may play major and important roles in this phenomenon by preceding the successful development of a single bud to the state of a functional zooid, while eliminating all other co-initiations within a single isolated fragment. Detailed depiction of cell signaling repertoire and networks within WBR phenomenon needs to be addressed in further controlled studies. Above results provide the first directions into the molecular machinery dictating *B. leachi *WBR, and highlight the importance and benefits of employing EST-based genome-wide analyses.

## Conclusion

Screening of an EST library from early stages of whole body regeneration in the colonial chordate *Botrylloides leachi *revealed 76 genes, belonging to Notch/Delta, Protein kinases, JAK/STAT, nuclear receptors, Ras oncogene family members, zinc-finger family members, GPCRs and TGF-β signaling. Together, with RT-PCR analysis we corroborate the involvement of these signaling pathways during whole body regeneration. We broadened our search by detailing the expression pattern of seven representative genes from the cell-signaling category and documented their expression pattern during whole body regeneration. The possible participation of these signaling pathways in other regeneration scenarios of diverse model systems as compared to the urochordates' whole body regeneration process, may disclose common and basic processes for development and regeneration in multicellular organisms.

## Methods

### Animal husbandry

*B. leachi *colonies were collected from shallow waters along the Israeli Mediterranean coast and carefully peeled off the underlying surfaces of stones by industrial razor blades. To minimize tissue damage, each peeled material included an attached thin layer of the calcareous substrate. Isolated colonies were individually tied with fine threads onto 5 × 7.5 cm glass slides and cultured in 17-liter tanks of standing seawater system, as described earlier [37]. Within several days under these culture conditions, the colonies glided or partially glided from their original calcareous substrate onto the glass slides. This movement involved repeated ampullar-contractions and expansions. Colonies and their substrates were cleaned weekly with industrial razor blades and fine brushes.

### Regeneration assay

Under a dissecting microscope, marginal ampullae and fragments of blood vessels were separated from colonies growing on glass slides, using an industrial razor blade and a fine tungsten needle. Then, dissected colonies were removed from the experimental slides and tied onto other slides. The leftover blood vessel fragments were cut into smaller fragments using a fine tungsten needle and were left to regenerate in 17-liter tanks. Fragments were monitored daily under a dissecting microscope, and photographed with a Supercam camera (Applitec, Holon, Israel).

### Analysis of endogenous transcripts by RT-PCR

Total RNA was isolated from fragments of regenerating blood vessels with RNeasy Mini or Midi kits (Qiagen, Valencia CA, USA) as template. First strand cDNA was synthesized by first strand DNA synthesis kit (Fermentas). The PCR amplification was performed using designed sets of primers (Operon; Table 1). Cytoplasmic actin primers were added to all samples, serving as the reference gene. The PCR reaction was carried out for 30 cycles (95°C, 1 min, 55–60°C, 1 min, 72°C, 1 min) followed by additional 10 minutes at 72°C. PCR products were analyzed in 1.5% agarose/EtBr gel alongside a DNA marker.

### Isolation of the *Bl-Rap1A*, *Bl-Cnot*, *Bl-Slit*, *Bl-STAT*, *Bl-Rab-33*, *Bl-Bax inhibitor *and *Bl-Mnk *homologues from *B. leachi*

178 bp, 469 bp, 453 bp, 454 bp, 316 bp, 578 bp, 475 bp, fragments corresponding respectively to *Bl-Rap1A, Bl-Cnot, Bl-Slit, Bl-STAT, Bl-Rab-33, Bl-Bax inhibitor *and *Bl-Mnk *were amplified from cDNA of regenerating blood vessels using sequence specific oligonucleotide primers. Forward: CTGTTAGGGCTGAGCATCATAG; reverse: TGGCAAGACAATGGAATAACTG for *Bl-Rap1A*. Forward: TTCTCCGTAACGATTCAACTTG; reverse: GGGTATTTGTCATGTTTCTTGC for *Bl-Cnot*. Forward: GGTTAATCGGCAGTCGCAAAG; reverse: GGCCTCAATAAATTACGTGTGC for *Bl-Slit*. Forward: AGACTCAGCTCCGCGTTTG; reverse: AATCATTTGGCAGAACGGAC for *Bl-STAT*. Forward: CGCAATATTAGTGTCCACATGG, reverse; TCCTACTCGAACTGAAGCCAC for *Bl-Rab-33*. Forward: AAATGAAATCTATGGCACAGCG; reverse: TGACTGCCTTTATGACAACCTC for *Bl-Bax inhibitor*. Forward: ATGTGGTGAGGATTGTGGATG; reverse: AATCGTGAATGTGGCTGAAGAC for *Bl-Mnk*. PCR products were cloned into Pdrive vector using Qiagen PCR cloning kit.

### In situ hybridization

*B. leachi *colonies, colonial fragments containing blood vessels, and regenerating fragments were fixed overnight in 4% paraformaldehyde, dehydrated in 70% methanol, embedded in paraffin, and cut into 5-μm sections. All seven clones were used to obtain sense and antisense DIG-labeled RNA probes that were synthesized using the DIG RNA labeling kit (SP6/T7, Roche Molecular Biochemicals, ). Hybridization of probes to tissue sections was performed according to Breitschopf [38] for paraffin-embedded tissue. DIG-labeled RNAs on samples were revealed using anti-DIG antibody (Roche). Samples were observed with the Leica DMIRE2 inverted microscope and photographed with a Leica FX300 camera.

## Authors' contributions

YR developed the EST library, performed the regeneration experiments, including the RT-PCR analysis and *in-situ *hybridization. YR, BR and RR analyzed the data. YR, BR and RR were involved in drafting the manuscript.

## Supplementary Material

Additional file 1 **Table S1.** Expressed gene products during early phases in *B. leachi *WBR that belong to cell communication and signaling and the top homologous match for each sequence as revealed by Blast analysis. The table provided data on the expressed genes from the EST library that belong to the cell communication and signaling category. Click here for file
