# Supplementary material for: Cell signaling and transcription factor genes expressed during whole body regeneration in a colonial chordate
Source: BMC Dev Biol. 2008 Oct 12;8:100. doi: 10.1186/1471-213X-8-100 (PMC2576188; doi:10.1186/1471-213X-8-100)
Supplement: Additional file 1 — Table S1. Expressed gene products during early phases in B. leachi WBR that belong to cell communication and signaling and the top homologous match for each sequence as revealed by Blast analysis. The table provided data on the expressed genes from the EST library that belong to the cell communication and signaling category. [file 1471-213X-8-100-S1.pdf]

Table S1. Expressed gene products during early phases in *B. leachi* WBR that belong to cell communication and signaling and the top homologous match for each sequence as revealed by Blast analysis.

Seq. No	Genebank Accession No	SwissProt Homologue ID	Gene identity	Organism	*E*-value	No of hits
1	**EW713271**	Q1MTB1_BRARE	milk fat globule-EGF factor 8 protein	*D. rerio*	5e-24	11
2	**EW713277**	Q7TQ50_MOUSE	Transmembrane receptor Notch1	*M. musculus*	4e-09	8
3	**EW713273**	Q6VEJ7_OREMO	Receptor for activated PKC	*O. mossambicus*	3e-77	7
4	**EW713274**	SOMA_SPAAU	Somatotropin precursor	*S. aurata*	1e-102	6
5	**EW713272**	Q1RL58_CIOIN	Zinc finger protein	*C. intestinalis*	2e-09	3
6	**EW713275**	Q28GN8_XENTR	BAX inhibitor 1	*X. tropicalis*	2e-48	3
7	**EW713276**	Q5R2J6_TRISI	Zinc finger containing transactivation factor Sp5	*T. sinensis*	1e-09	2
8	**EW713270**	Q5RHV3_BRARE	vertebrate dual-specificity tyrosine-(Y)-phosphorylation regulated kinase	*D. rerio*	8e-31	2
9	**EW713278**	1433F_RAT	14-3-3 protein eta	*R. norvegicus*	1e-31	2
10	**EW713279**	Q1RL58_CIOIN	Zinc finger protein	*C. intestinalis*	1e-60	1
11	**EW713280**	Q54KX0_DICDI	GATA-binding transcription factor	*D. discoideum*	5e-30	1
12	**EW713281**	O74649_CRYNE	Rho1 GTPase	*C. neoformans*	2e-07	1
13	**EW713282**	Q5PQK8_RAT	Transforming growth factor beta regulated gene	*R. norvegicus*	3e-12	1
14	**EW713283**	Q6T388_FUCDI	Rho family GTPase	*F. distichus*	1e-16	1
15	**EW713284**	Q1RL34_CIOIN	Zinc finger protein	*C. intestinalis*	1e-09	1
16	**EW713285**	IMB1_RAT	Importin beta-1 subunit	*R. norvegicus*	4e-11	1
17	**EW713286**	Q8WUW5_HUMAN	PRMT1 protein	*H. sapiens*	1e-62	1
18	**EW713287**	Q5CZY5_MOUSE	Cnot2 protein	*M. musculus*	4e-46	1
19	**EW713288**	GPR89_BOVIN	Protein GPR89A	*B. taurus*	2e-30	1
20	**EW713289**	MKNK2_XENTR	MAPK-interacting serine/threonine-protein kinase 2	*X. tropicalis*	2e-36	1
21	**EW713290**	DNJA3_MOUSE	DnaJ protein Tid-1	*M. musculus*	1e-29	1
22	**EW713291**	Q659T9_CIOIN	Putative serine protease 7	*C. intestinalis*	1e-11	1
23	**EW713292**	Q5VNG7_ORYSA	Putative DsPTP1 protein	*O. sativa*	4e-20	1
24	**EW713293**	RDH14_HUMAN	Retinol dehydrogenase 14	*H. sapiens*	0.069	1
25	**EW713294**	Q91YS9_MOUSE	Eph receptor B3	*M. musculus*	1e-07	1
26	**EW713295**	Q5MD60_HUMAN	Mitogen-activated protein kinase kinase kinase kinase 4 isoform	*H. sapiens*	1e-44	1
27	**EW713296**	CREL1_MOUSE	Cysteine-rich with EGF-like domain protein 1 precursor	*M. musculus*	1e-07	1
28	**EW713297**	Q86KZ2_DICDI	TFIIH	*D. discoideum*	6e-27	1
29	**EW713298**	Q2M1L3_HUMAN	G protein-coupled receptor 133	*H. sapiens*	3e-10	1
30	**EW713299**	Q2KIV3_BOVIN	Similar to zinc responsive protein ZD7	*B. taurus*	3e-12	1
31	**EW713300**	Q6P0P0_HUMAN	Methyl-CpG binding domain protein	*H. sapiens*	4e-10	1
32	**EW713301**	Q7ZWC1_BRARE	Integrin beta 4 binding protein	*D. rerio*	6e-38	1
33	**EW713302**	Q9Y1V3_POLMI	Tunicate retinoic acid-inducible protease	*P. misakiensis*	1e-38	1
34	**EW713303**	AKT1_BOVIN	RAC-alpha serine/threonine-protein kinase	*B. taurus*	1e-13	1

35	**EW713304**	Q1RPV5_CIOIN	Zinc finger protein	*C. intestinalis*	5e-28	1
36	**EW713305**	Q4H3E0_CIOIN	Transcription factor protein	*C. intestinalis*	1e-37	1
37	**EW713306**	Q8N5U6_HUMAN	Ring finger protein 10	*H. sapiens*	2e-27	1
38	**EW713307**	Q8AWF9_BUFBG	Zinc finger protein Yan	*B. gargarizans*	6e-12	1
39	**EW713308**	Q4H2R0_CIOIN	Ci-STAT-b protein	*C. intestinalis*	1e-79	1
40	**EW713309**	Q9BYI2_HUMAN	G kinase anchoring protein 1	*H. sapiens*	1e-04	1
41	**EW713310**	Q6Y9Q3_BRAFL	TATA-binding protein isoform 2	*B. floridae*	2e-29	1
42	**EW713311**	Q25058_HELER	Fibropellin Ia	*H. erythrogramma*	3e-22	1
43	**EW713312**	Q86SC9_CIOIN	IQ motif containing GTPase activating protein	*C. intestinalis*	3e-28	1
44	**EW713313**	Q86SC9_CIOIN	IQ motif containing GTPase activating protein	*C. intestinalis*	6e-13	1
45	**EW713314**	RBCC1_MOUSE	RB1-inducible coiled-coil protein	*M. musculus*	8e-11	1
46	**EW713315**	Q5CZT6_BRARE	Zgc:113125	*D. rerio*	1e-27	1
47	**EW713316**	Q3TNK3_MOUSE	glutathione peroxidase 7	*M. musculus*	2e-13	1
48	**EW713317**	WDR48_MOUSE	WD repeat protein 48	*M. musculus*	1e-06	1
49	**EW713318**	Q1RLC8_CIOIN	Zinc finger protein	*C. intestinalis*	2e-12	1
50	**EW713319**	Q1RLF8_CIOIN	Zinc finger protein	*C. intestinalis*	6e-04	1
51	**EW713320**	Q800Y7_MELGA	Hepatocyte growth factor activator	*M. gallopavo*	5e-17	1
52	**EW713321**	Q63274_RAT	Kallikrein	*R. norvegicus*	3e-10	1
53	**EW713322**	Q6UKZ2_MOUSE	Serpinb3b	*M. musculus*	3e-05	1
54	**EW713323**	HTK16_HYDAT	Tyrosine-protein kinase HTK16	*H. attenuata*	7e-24	1
55	**EW713324**	Q4H323_CIOIN	Transcription factor protein	*C. intestinalis*	2e-08	1
56	**EW713325**	TRIB2_HUMAN	Tribbles homolog 2 (TRB-2)	*H. sapiens*	2e-22	1
57	**EW713326**	Q9U6N1_MUSDO	Cytochrome P450	*M. domestica*	2e-07	1
58	**EW713327**	Q1WDE8_PINPS	Rab1	*P. pinaster*	6e-09	1
59	**EW713328**	Q2TBV5_BOVIN	TFIIH	*B. taurus*	5e-25	1
60	**EW713329**	Q6J1M0_XENLA	Rap 1A2 GTPase	*X. laevis*	4e-16	1
61	**EW713330**	Q17B38_AEDAE	Insl3/relaxin receptor	*A. aegypti*	6e-09	1
62	**EW713331**	SPI1_PIG	Transcription factor PU.1	*S. scrofa*	1e-09	1
63	**EW713332**	ALDH9_POLMI	Aldehyde dehydrogenase 9	*P. misakiensis*	1e-46	1
64	**EW713333**	HMCN1_HUMAN	Hemicentin-1 precursor (Fibulin-6)	*H. sapiens*	1e-16	1
65	**EW713334**	RAB33_CIOIN	Ras-related protein Rab-33	*C. intestinalis*	9e-59	1
66	**EW713335**	Q6WMS9_BRABE	Interferon gamma-inducible protein	*D. rerio*	2e-29	1
67	**EW713336**	Q7ZYY5_GASAC	Signal peptidase 25 kDa subunit	*G. aculeatus*	8e-29	1
68	**EW713337**	TIMP2_CAVPO	Tissue inhibitor of metalloproteinases	*C. porcellus*	7e-07	1
69	**EW713338**	Q8R9K4_THETN	Putative translation initiation inhibitor	*T. tengcongensis*	1e-16	1
70	**EW713339**	SLIT3_HUMAN	Slit homolog 3 protein precursor	*H. sapiens*	9e-11	1
71	**EW713340**	Q91WU4_MOUSE	Transmembrane and coiled-coil domains 4	*M. musculus*	1e-24	1
72	**EW713341**	Q5PP79_9ALPH	Secreted glycoprotein gG precursor	*S. herpesvirus*	4e-04	1
73	**EW713342**	WIPI4_BRARE	WIPI-4	*D. rerio*	1e-23	1
74	**EW713343**	Q2DQL6_9DELT	ATP-NAD kinase	*G. uraniumreducens*	3e-21	1
75	**EW713344**	Q8AXP0_CYNPY	Receptor protein Notch1	*C. pyrrhogaster*	6e-22	1
76	**EW713269**	Q9H9M5_HUMAN	thyroid hormone receptor-associated protein	*H. sapiens*	1e-16	1
